# Commentary: Emotion effects on implicit and explicit musical memory in normal aging

**DOI:** 10.3389/fpsyg.2017.02208

**Published:** 2017-12-14

**Authors:** Nicola Mammarella

**Affiliations:** Psychological Sciences, Health and Territory, Università degli Studi “G. d'Annunzio” Chieti - Pescara, Chieti, Italy

**Keywords:** implicit memory, emotion, aging, positivity bias, music cognition

Implicit emotional memory refers to unconscious retrieval of past emotional experiences (e.g., Graf and Schacter, 1985). The study by Narme et al. (2016) is one of the first to examine age-related changes in implicit emotional memory using unfamiliar emotional melodies that varied in terms of valence (positive: peaceful and happy; negative: sad and fearful) and arousal (high or low). Implicit memory was assessed with a preference task in which participants rated their liking of previously studied or new melodies. In particular, 113 participants were involved, 60 younger adults (58.3% women, mean age 23.6) and 53 older adults (68% women, mean age 75.3). These two groups of participants first heard a list of musical excerpts and then they had to indicate whether each musical piece had a fast or slow tempo. Half of melodies were played twice and the other half six times. Subsequently, participants were asked to rate how much they liked each melody using a 10-point scale (from 1 = *I do not like it at all* to 10 = *I like it a lot*) for old and new excerpts.

Although there was a slight decrease in implicit memory, results show that exposure effects, that is, higher liking ratings for old melodies compared with new ones, occurred in both groups. Most importantly, the authors found no particular aging effects on emotional implicit memory for music, for example, in terms of the classical age-related positivity effect (e.g., Mather, 2012). In fact, there were no stronger exposure effects for positive melodies.

Given the general preservation of implicit memory (e.g., Jelicic, 1995; LaBar et al., 2005; Ward et al., 2013) coupled with the well-established positivity effect in the aging mind (e.g., Mather, 2012; Di Domenico et al., 2015), these data are intriguing. The rationale being that studying emotional memory-music interaction in aging is complex due to differences between emotion processing, in general, and the emotional processing of music, in particular.

From the emotion processing literature' point of view, one viable account for explaining the absence of positivity effects may be linked to the level of stimulus familiarity at test. This may be particularly true in the presence of musical excerpts and lead to a consequent modulation of valence effects. There are studies, in fact, showing how positive valence is typically linked to processing fluency and feelings of familiarity such as the more fluently people process an event, the more positive is their response (e.g., Winkielman and Cacioppo, 2001; Berridge and Winkielman, 2003). Therefore, if participants assign a certain level of familiarity to the target at test, they may tend to react in a positive manner, even in the presence of negative events. Consequently, results should be taken with caution. The general findings that participants liked the excerpts they heard six times more than those they heard twice and that no valence effects occurred especially with high arousing stimuli in the older adults' group may point to this direction.

Moreover, the authors stated that the absence of a positivity effect in implicit memory in older adults suggests that the positivity effect stems from top-down processes. This conclusion did not take into account studies (e.g., Hess et al., 2004) showing that older adults' memory performance could be unconsciously influenced by the activation of negative and positive aging stereotypes. In particular, older adults' performance was significantly lower in the negative priming condition than in the positive. In addition, age differences were significantly reduced following the positive prime.

Another relevant aspect of the study is the potential for explicit contamination. As suggested by Mulligan (2011), when given an implicit memory test, participants may become aware of the relationship between study and test and consequently engage in intentional retrieval. The authors did not use, for instance, any post-test questionnaire to determine if participants were aware of the connection between the study and test phases of the experiment (the authors themselves acknowledged this as a limitation).

Finally, another aspect that need further consideration refers to mood-congruent effects in implicit memory, that is the finding that emotional information congruent with current mood is more likely to be recalled than information incongruent with current mood. For example, Gaddy and Ingram (2014) found that depressive groups exhibited preferential implicit recall of negative information and non-depressed groups exhibited preferential implicit recall of positive information. Although older adults were screened for depression, it is possible that musical excerpts themselves acted as a mood induction technique and later influenced implicit emotional memory.

From the emotional processing of music's perspective, one can argue that the distinction between positive and negative valence typically found in emotion research may not apply to a full extent to music. In fact, music processing relies on a series of cognitive skills (e.g., decoding of pitch, rhythm, timbre, and processing of sequential elements that may convey emotions) and a series of neural networks (e.g., amygdala, nucleus accumbes, posterior cingulate cortex) that show age-related differences (from newborns to older adults, e.g., Perani et al., 2010) and are strongly modulated by lifelong experiences (e.g., Spada et al., 2014). Consequently mental representations of an auditory scene and the corresponding emotional meaning may reflect the interaction between top-down and bottom-up processes.

In sum, while Narme et al.'s data are some of the first to focus on implicit emotional memory in aging, it has to be acknowledged that the study of emotional memory-music interaction is complex. For example, a familiarity and fluency-based interpretation of the interaction between implicit memory and emotion cannot be ignored. In addition, controlling for explicit contamination (e.g., Fairfield et al., 2015) is highly relevant for directing future research in this new area, especially in the aging mind. However, researchers in emotion-memory interaction must be aware that emotion processing findings and explanations cannot be easily applied to the context of music processing.

## Author contributions

The author confirms being the sole contributor of this work and approved it for publication.

### Conflict of interest statement

The author declares that the research was conducted in the absence of any commercial or financial relationships that could be construed as a potential conflict of interest.
